# Impact of drug coated balloon on left anterior descending artery stenosis at proximal to myocardial bridge

**DOI:** 10.3389/fcvm.2026.1830690

**Published:** 2026-08-24

**Authors:** Yuqing Jia, Yangfeng Xie, Ying Li, Huaipeng Zhang, Yanfen Li, Weiming Li, Chuang Li, Yixing Yang, Wenhao Jia, Wenbo Han

**Affiliations:** 1 Department of Cardiology, Heze Municipal Hospital, Heze, China; 2 Department of Cardiology, Dongzhimen Hospital, Beijing University of Chinese Medicine, Beijing, China; 3 Department of Cardiology, Beijing Chao-Yang Hospital, Capital Medical University, Beijing, China; 4 Department of Cardiology, The Third Affiliated Hospital, Beijing University of Chinese Medicine, Beijing, China

**Keywords:** coronary heart disease, *de novo* lesion, drug-coated balloon, myocardial bridge, percutaneous coronary intervention

## Abstract

**Background:**

Drug-coated balloons (DCB) represent a novel therapeutic approach for treating native coronary artery disease. However, the clinical outcomes of DCB in patients with left anterior descending (LAD) artery stenosis proximal to a myocardial bridge (MB) remain unclear. This study aims to investigate the effects of DCB in patients with LAD artery stenosis proximal to MB.

**Method:**

This retrospective cohort study included consecutive patients with LAD artery stenosis proximal to MB who underwent DCB or drug-eluting stent (DES) procedures between January 2020 and July 2021. The DCB group comprised 30 patients treated with DCB alone, while the DES group included 105 patients treated with DES alone. Major adverse cardiac events (MACEs) during a median follow-up of 507 days (IQR 391.5–622.0 days) were defined as a composite of unstable angina requiring hospitalization, acute myocardial infarction, cardiac death, and target lesion revascularization (TLR).

**Results:**

Both groups exhibited generally comparable baseline clinical and lesion characteristics, except for age and MB systolic compression. Over a median follow-up of 507 days (IQR 391.5–622.0 days), Kaplan–Meier survival analysis revealed no statistically significant difference in MACE-free survival rates between the two groups (73.96%, 95% CI 55.8–98.0 vs. 82.49%, 95% CI 73.9–90.8; Log-rank *P* = 0.678). Cox proportional regression analysis showed no statistically significant difference in MACEs between DCB and DES patients (HR = 1.58, 95% CI = 0.56–4.45, *P* = 0.383).

**Conclusion:**

In this exploratory retrospective study, we did not detect a statistically significant advantage of DCB over DES in treating LAD stenosis proximal to MB. Given the limited sample size, these findings should be considered preliminary and do not support DCB as a reasonable alternative to DES. Larger prospective studies are warranted to validate the potential role of DCB in this specific anatomical setting.

## 1 Introduction

Myocardial bridge (MB) is a congenital coronary artery anomaly characterized by a segment of an epicardial coronary artery passing through the myocardial tissue (1). The left anterior descending artery (LAD) has the highest incidence of MB, with most cases having a positive prognosis. However, some patients experience adverse cardiovascular events, such as acute coronary syndrome (2, 3). Severe pathological stenosis can develop in the proximal segment of the MB due to blood flow shear forces, necessitating further interventional therapy (4, 5). The primary treatment for stenosis proximal to a MB is stent implantation; however, complications such as in-stent restenosis are challenging to avoid due to the proximity of the lesion to the MB (6, 7).

Drug-coated balloons (DCB) have gained popularity in clinical practice. Unlike drug-eluting stents (DES), DCBs leave no polymer matrix or metal mesh behind, reducing the risk of intimal inflammation and thrombosis while avoiding metal implants in the body. This has a positive clinical impact on treating lesions, including bifurcated coronary lesions and small coronary vessels (8, 9). However, the clinical outcomes of DCB for stenosis proximal to a MB remain unclear. Given the limitations of DES in this challenging anatomical subset, this study aimed to compare clinical outcomes between DCB and DES in patients with LAD stenosis proximal to an MB.

## 2 Material and methods

### 2.1 Study population

This study included patients from Beijing Chao-Yang Hospital with LAD artery stenosis proximal to a MB from January 2020 to July 2021. Diagnosis of MB was based on a segment being compressed during systole by surrounding muscle contraction and recovering fully or partially during diastole (10). Proximal atherosclerotic lesions of MB were defined as significant stenosis (over 70%), as assessed by quantitative coronary angiography (QCA). Patients aged ≤85 years and with a target vessel diameter >2.0 mm were included, while those with left main vessel lesions or prior coronary artery bypass graft surgery were excluded. This human study was approved by the Institutional Review Board (2020-KY025), conducted in accordance with the Declaration of Helsinki, and registered in the Chinese Clinical Research Registry (No. ChiCTR2200066421).

### 2.2 Procedures and medication

Before coronary angiography (CAG) and percutaneous coronary intervention (PCI), all patients received 300 mg of aspirin and a loading dose of either 300 mg of clopidogrel or 180 mg of ticagrelor. The radial artery was initially evaluated, and the surgeon decided on the use of glycoprotein IIb/IIIa receptor inhibitors. After discharge, dual antiplatelet therapy followed global recommendations (11). The interventional technique (DCB or DES) was chosen by an experienced interventionist team following diagnostic coronary angiography. The DCB group received routine pre-dilatation using a standard balloon. Cutting balloons or non-compliant scoring balloons [non-slip element (NSE)] were utilized at the operators’ discretion to obtain an acceptable lumen increase. DCB diameter was selected with a 1:1 ratio to the reference vessel diameter as determined by QCA. All percentage stenoses were measured by quantitative coronary angiography (QCA) as described in Section 2.3. After pre-expansion processing, the applicable conditions of DCB are:(a) TIMI blood flow grade III; (b) Residual stenosis ≤30% (c) Dissection below Type C. Specifically: TIMI < III means grades 0, I, or II (no flow, minimal flow with stagnation, slow flow). Residual stenosis >30% is diameter stenosis >30%. Dissection type C - F includes transmural (C), spiral (D), persistent filling defect (E), or occlusion (F). Bailout stenting with DES was performed if any of these occurred. DCB should be delivered to the lesion within 2 min and given sufficient dilation time (≥30 s) and nominal pressure (7–8 atm). Applicable conditions for DES-alone or bailout stenting were: (a) TIMI flow < grade III; (b) Residual stenosis >30%; (c) Dissection type C-F. All decisions were independently evaluated by two senior interventional physicians. Patients were implanted with Paclitaxel-Coated Balloons (PCB, SeQuent Please, B. Braun, Melsungen, Germany) during PCI in DCB group. In DES group, patients were implanted with new generation DES, such as, Zotarolimus-eluting stent (ZES, Resolute Integrity, Medtronic, Galway, Ireland), Everolimus-eluting stent (EES, Promus PREMIER, Boston Scientific, Galway, Ireland). In the DCB group, dual antiplatelet therapy (DAPT) was de-escalated to single antiplatelet therapy after 3 months. Conversely, DES patients received DAPT for 1 year before transitioning to monotherapy. Based on medical records and follow-up interviews, no patient was found to have deviated from the planned DAPT regimen.

### 2.3 Quantitative coronary angiographic analysis

The percentage of diameter stenosis, percentage of MB compression, MB length and lesion length were analysed by automated edge-detection system (Allura Xper FD20, Philips, Netherlands) before and after angiography.

### 2.4 Clinical follow-up and study end points

Patients were followed up via phone or outpatient clinic interviews every three months after PCI until the administrative censoring date of May 22, 2022. Follow-up was conducted for major adverse cardiac events (MACEs), defined as unstable angina requiring hospitalization, acute myocardial infarction, cardiac death, and target lesion revascularization (TLR). MACE-free survival was defined as the time from the index procedure to the first occurrence of any of these events. MACEs were adjudicated by two cardiologists based on hospital medical records and follow-up telephone interviews. Due to the retrospective design, a blinded independent endpoint committee was not used.

### 2.5 Statistical analysis

The analysis was performed using R programming language (version 3.6.1) and EmpowerStats (version 4.0). Normality of continuous variables was assessed using the Shapiro-Wilk test. Normally distributed variables are presented as mean ± standard deviation (SD) and were compared using the unpaired Student's t test. Non-normally distributed variables are presented as median (interquartile range, IQR) and were compared using the Mann-Whitney U test. Categorical variables were compared using the chi-square test. Kaplan-Meier survival analysis was used to generate cumulative event-free survival curves. Cox proportional regression analysis, adjusted for confounding baseline variables, was used to evaluate the potential impact of intervention on MACEs, with hazard ratios (HRs) and 95% confidence intervals (CIs). Multivariate Cox regression was chosen for covariate adjustment instead of propensity-score-based methods, given the small sample size. A two-sided *P* value < 0.05 was considered statistically significant.

## 3 Results

**Figure 1:**
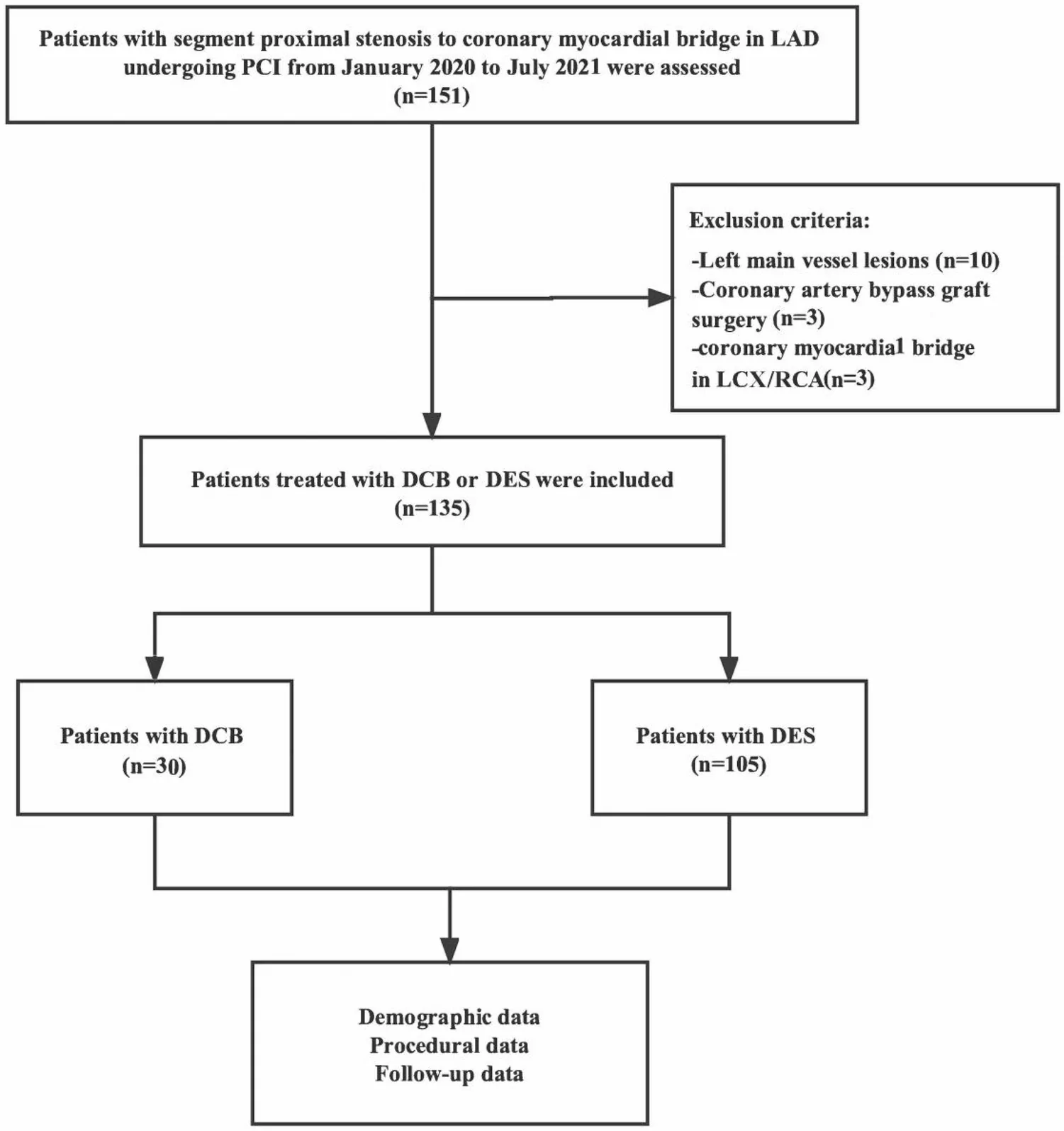
Flow of patient inclusion in the study. DCB, drug-coated balloon; DES, drug-eluting stent; LAD, left anterior descending coronary artery; PCI, percutaneous coronary intervention.

From January 2020 to July 2021, a total of 135 consecutive patients were enrolled, with 30 receiving DCB and 105 receiving DES (Figure 1). No patient in the DCB group required bailout stenting. The average age was 63.38 ± 9.57 years, with 103 (76.3%) being male. Baseline characteristics were generally comparable between the two groups, although the DCB group was older (66.40 ± 9.07 vs. 62.51 ± 9.58 years). Table 1 displays baseline demographic and clinical information.

**Table 1:** Baseline patient characteristics.

Characteristic	DCB group (*n* = 30 )	DES group (*n* = 105 )	*P*
Male	22 (73.33%)	81 (77.14%)	0.665
Age (years)	66.40 ± 9.07	62.51 ± 9.58	0.050
BMI (kg/m^2^)	26.22 ± 2.04	25.33 ± 3.23	0.158
Smoking	16 (53.33%)	60 (57.14%)	0.711
Hypertension	22 (73.33%)	60 (57.14%)	0.109
Prior PCI	5 (16.67%)	28 (26.67%)	0.261
Stroke	1 (3.33%)	15 (14.29%)	0.102
LDL-C (mmol/L)	2 (1.53, 2.88)	2.26 (1.67, 2.85)	0.604
ALT (U/L)	21 (16.00, 35.25)	19 (14.00, 32.00)	0.217
Cr (umol/L)	68.05 (56.75, 73.58)	70.30 (58.55, 79.40)	0.440
HsCRP (mg/L)	1.12 (0.40, 5.06)	1.3 (0.50, 3.48)	0.749
HbA1c (%)	6.05 (5.55, 7.65)	6.1 (5.7, 7.15)	0.769
LVEF (%)	66 (58.00, 73.00)	66 (61.00, 70.50)	1.000
Admission diagnosis			
UA	22 (73.33%)	68 (64.76%)	0.500*
NSTEMI	5 (16.67%)	19 (18.10%)	
STEMI	3 (10.00%)	18 (17.14%)	

*P value obtained from Fisher's exact test.

ALT, alanine aminotransferase; BMI, body mass index; Cr, creatinine; DCB, drug-coated balloon; DES, drug-eluting stent; LDL-C, low-density lipoprotein cholesterol; LVEF, left ventricular ejection fraction; NSTEMI, non-ST elevated myocardial infarction; PCI, percutaneous coronary intervention; STEMI, ST elevated myocardial infarction; UA, unstable angina pectoris.

The lesion length in the DCB group was 26.5 (18.0, 29.35) mm, while in the DES group it was24.0 (17.5, 35.0) mm (*P* = 0.605). There was no significant difference in MB length between the two groups 20.8 (13.73, 30.28)mm for DCB vs. 20.87 (17.08, 28.06) mm for DES, *P* = 0.797), nor in the distance from stenosis to MB [7.42 (5.13, 9.29) for DCB vs. 7.16 (4.39, 9.48) for DES, *P* = 0.935]. However, the DES group exhibited more severe stenosis [90 (80.0, 95.0)% vs. 90 (85.0, 97.0)%, *P* = 0.153] and less MB systolic compression [50 (47.5, 72.5)% vs. 50 (40.0, 55.0)%, *P* = 0.015] (Table 2).

**Table 2:** Angiographic characteristics.

Characteristic	DCB group (*n* = 30 )	DES group (*n* = 105 )	*P*
Lesion stenosis (%)	90 (80.00,95.00)	90 (85.00,97.00)	0.153
Lesion length (mm)	26.5 (18.00,29.35)	24 (17.50,35.00)	0.605
MB length (mm)	20.8 (13.73,30.28)	20.87 (17.08,28.06)	0.797
MB systolic oppression (%)	50 (47.50,72.50)	50 (40.00,55.00)	0.015
Distance from stenosis to MB (mm)	7.42 (5.13,9.29)	7.16 (4.39,9.48)	0.935
DES diameter (mm)	—	3.22 ± 0.38	—
DES length (mm)	—	30.64 ± 13.57	—
DCB diameter (mm)	2.80 ± 0.35	—	—
DCB length (mm)	28.57 ± 11.75	—	—

DCB, drug-coated balloon; DES, drug-eluting stent; MB, myocardial bridge.

**Figure 2:**
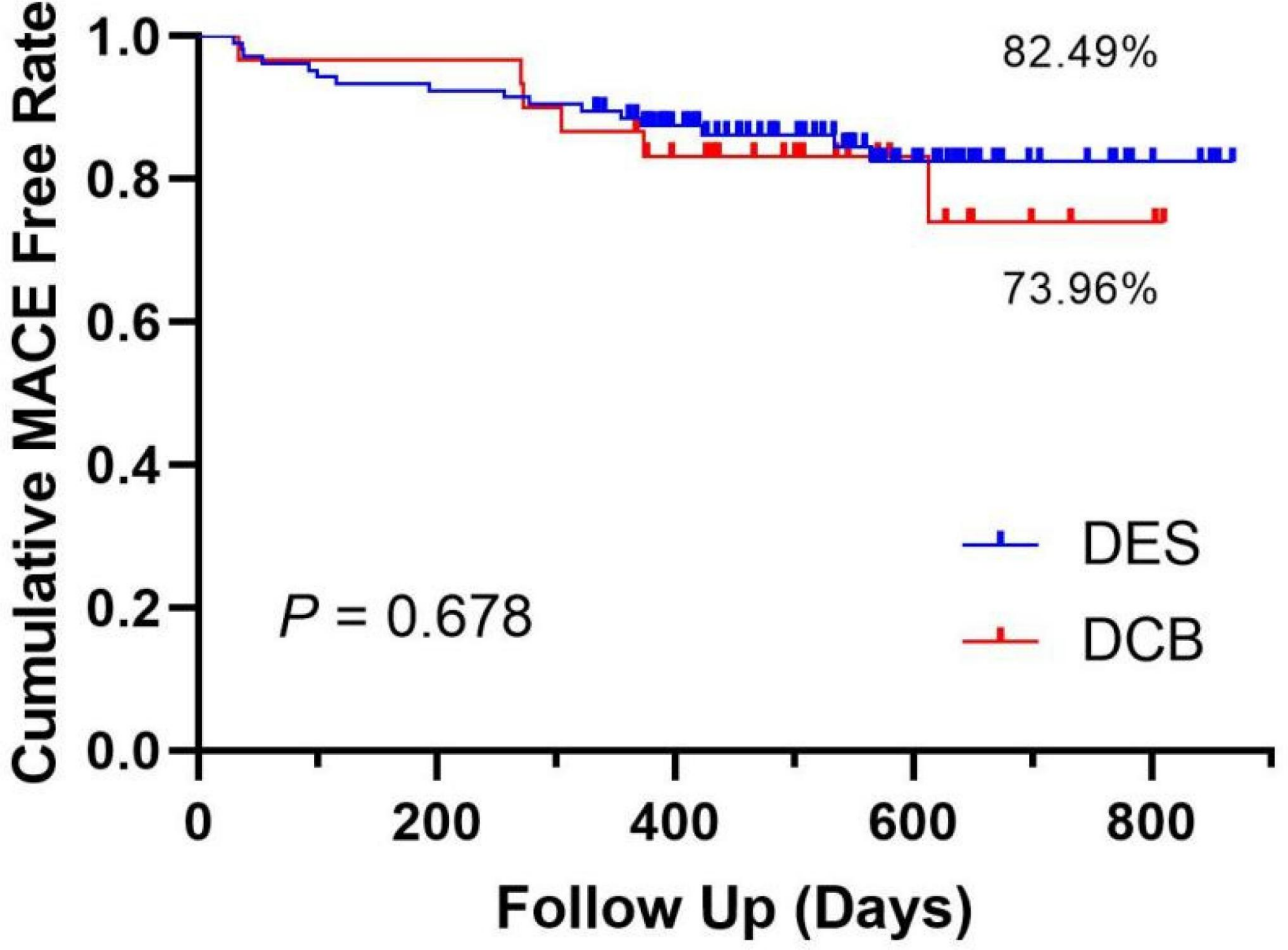
Kaplan–meier curves for MACE-free survival during a median follow-up of 507 days (IQR 391.5−622.0 days; censored as of May 22, 2022). Kaplan–Meier survival analysis revealed no statistically significant difference in MACE-free survival rates between the two groups (log-rank *P* = 0.678). DCB, drug-coated balloon; DES, drug-eluting stent.

During a median follow-up of 507.0 days (IQR 391.5–622.0 days; censored as of May 22, 2022), 21 (15.6%) patients experienced composite outcomes. Kaplan–Meier survival analysis revealed no statistically significant difference in MACE-free survival rates between the two groups (DCB: 73.96%, 95% CI 55.8–98.0; DES: 82.49%, 95% CI 73.9–90.8; Log-rank *P* = 0.678, Figure 2).No statistically significant differences were observed in composite endpoints such as acute myocardial infarction, acute heart failure, or unstable angina between the two groups. Table 3 lists the clinical events. A multivariate Cox proportional regression model was employed to adjust for potential confounders (Table 4) and outcomes showed no statistically significant differences, indicated that DCB did not impact MACEs.

**Table 3:** Clinical events.

Characteristic	DCB group (*n* = 30 )	DES group (*n* = 105 )	*P*
MACE	6 (20.00)	15 (14.29)	0.446
Cardiac death	0 (0.00)	0 (0.00)	—
AMI	1 (3.33)	1 (0.95)	0.341
UA	5 (16.67)	14 (13.33)	0.643
TLR	0 (0.00)	0 (0.00)	—

Values are *n* (%).

AMI, acute myocardial infarction; DCB, drug-coated balloon; DES, drug-eluting stent; MACE, major adverse cardiac events; TLR, target lesion revascularization; UA, unstable angina.

**Table 4:** Multivariate cox regression analyses of MACE.

Variables	Hazard ratio (95%CI)	*P*
DCB (vs. DES)	1.58 (0.56–4.45)	0.383
Age	1.00 (0.95–1.05)	0.963
Male	0.55 (0.21–1.45)	0.225
BMI	0.94 (0.81–1.08)	0.368
Stroke	1.93 (0.51–7.27)	0.330
LDL-C	1.19 (0.75–1.91)	0.459
HbA1c	0.77 (0.50–1.17)	0.214

Model fit: likelihood ratio test *χ*^2^ = 14.2, *P* = 0.05; Harrell's C-index = 0.74 (SE = 0.05). In a sensitivity analysis adjusting for additional angiographic covariates, the HR for DCB vs. DES remained non-significant (HR = 1.27, 95% CI 0.44–3.68, *P* = 0.665).

BMI, body mass index; CI, conﬁdence interval; DCB, drug-coated balloon; DES, drug-eluting stent; LVEF, left ventricular ejection fraction; MACE, major adverse cardiac events. PCI, percutaneous coronary intervention.

## 4 Discussion

MB is a prevalent congenital abnormality found in approximately one-third of adults, often diagnosed through coronary angiography and computed tomography. Previous studies indicate that about 82% of MBs occur in the LAD, primarily in the proximal and middle segments (12, 13). While many patients remain asymptomatic, others may experience coronary heart disease due to the effects of direct compression and atherosclerosis in the LAD segment proximal to MB (5, 14). A meta-analysis, which involved 4,556 subjects (30.5% presented MB), showed that MB was associated with an increased risk of adverse cardiac events (OR = 1.71, 95% CI = 1.29–2.26, *P* = 0.0002), non-fatal myocardial infarction (OR = 3.17, 95% CI = 1.21–8.31; *P* = 0.02), and angina requiring hospitalization (OR = 2.31, 95% CI = 1.55–3.45; *P* < 0.0001), respectively, compared with subjects without MB (2).

Treating stenosis in the proximal segment of MB presents a clinical challenge, as coronary anatomy must be carefully considered. PCI with DES is a common approach, but it carries risks such as in-stent restenosis and other cardiovascular events. Studies have shown higher incidences of MACEs in patients receiving DES for significant stenosis proximal to MB compared with those without MB. According to one study by Zhang et al. (7), DES implantation for significant atherosclerosis stenosis in the segments proximal to MB have higher incidence of MACEs (HR = 1.781, 95% CI = 1.108–2.863; *P* = 0.017). Lee's study observed 551 patients with DES implantation, which were divided into MB group (proximal segment stenosis to MB) and non-MB group (6). The results showed that MACEs in MB group was significantly higher than that in non-MB group (18.1 vs. 9.8%, *P* = 0.024). These observations shows that MACEs of DES were likely to occur on stenosis at proximal to MB, and the mechanism may be related to endothelial dysfunction (15, 16).

DCBs have been increasingly used in treating stent restenosis, small-vessel disease and bifurcated disease recent years, and new indications are expanding, such as large-vessel disease, chronic total occlusion (CTO) lesions, diffuse coronary lesions, and acute coronary syndromes (17–19), which presents an opportunity for the management of stenosis at proximal to MB.

Myocardial bridges impose cyclic mechanical compression on the adjacent coronary segment, which may increase the risk of stent fracture, in-stent restenosis, and endothelial dysfunction when a rigid metal stent is implanted across or near the bridge (16, 20, 21). By leaving no metallic implant behind (22, 23), drug-coated balloons avoid the mechanical mismatch between a rigid stent and the dynamically contracting myocardial bridge, potentially reducing shear stress abnormalities and vessel wall irritation. Furthermore, the absence of a permanent polymer and metal platform may lower the risk of local inflammation and late thrombosis (24). Although direct evidence on drug uptake in segments proximal to MB is limited, studies have shown that paclitaxel can be effectively transferred to the vessel wall with a short exposure time (25), which may be sufficient to inhibit neointimal proliferation in such complex lesions. These mechanistic considerations provide a theoretical rationale for exploring DCB in this setting, but clinical evidence from our study does not support its advantage over DES.

In recent years, accumulating evidence has supported the clinical application of drug-coated balloons (DCB) for *de novo* coronary macrovascular lesions (≥3.0 mm) (23). The ongoing REVERSE trial (NCT05846893) is further evaluating DCB versus drug-eluting stents (DES) for macrovascular disease, with results anticipated to provide critical guidance for clinical practice. In our cohort, proximal MB lesions had vessel diameters of 2.80 ± 0.35 mm, and no statistically significant difference outcomes during a median follow-up of 507 days (IQR 391.5–622.0 days) was observed between DCB and DES. However, the REC-CAGEFREE I trial revealed that DCB with bailout stenting failed to achieve non-inferiority to DES for the 2-year device-oriented composite endpoint (DoCE) in uncomplicated coronary disease, underscoring remaining challenges for DCB in large-vessel interventions (26).

While intravascular imaging (IVUS/OCT) significantly optimizes procedural strategy and prognosis (27, 28), its utilization in this study was limited (8.89%, *n* = 12) due to resource constraints—consistent with China's national averages (IVUS: 8.1%; OCT: 1.5%, Statistics of the China Cardiovascular Health and Disease Report 2023). Nevertheless, our DCB approach for proximal MB lesions represented an early exploratory effort in China. Chinese Expert Consensus on the Clinical Application of Drug-Coated Balloons was updated in 2023, which also added the indication of DCB in Denovo lesions (29).

Several limitations should be acknowledged. First, the wide confidence interval and limited sample size preclude definitive conclusions despite the absence of a statistically significant difference. This was a retrospective, single-center study with a small sample size, particularly the DCB group (*n* = 30), which may have introduced potential selection bias. No *a priori* power calculation was performed; therefore, the study is underpowered to detect a 10% absolute difference in MACE-free survival during a median follow-up of 507 days (e.g., with 80% power, *α*=0.05), and the absence of a statistically significant difference should not be interpreted as equivalence. Baseline imbalances existed (older age and higher MB systolic compression in the DCB group). Although we adjusted for age and other covariates in the multivariate Cox regression model, residual confounding due to unmeasured or imprecisely measured variables may still exist. The rate of intravascular imaging was low (8.9%), mainly due to operator discretion and patient economic constraints, which may have introduced bias in lesion preparation and outcome assessment. Additionally, the examination of multiple secondary endpoints increases the risk of type I error. In addition, detailed DAPT adherence data were not systematically recorded due to the retrospective design, which is another limitation. Given these limitations, our findings are hypothesis-generating, suggesting that DCB may offer similar short-term clinical outcomes to DES in this lesion subset, but whether it confers any long-term advantage or benefits specific patient subgroups requires confirmation in larger prospective studies.

In conclusion, in this exploratory retrospective study, DCB did not demonstrate an advantage over DES in terms of MACE-free survival during a median follow-up of 507 days for treating LAD stenosis proximal to MB (HR = 1.58, 95% CI = 0.56–4.45). Based on these findings, there is no evidence to support DCB as a reasonable alternative to DES in this specific lesion subset. Future large-scale prospective studies are needed to further evaluate the potential role of DCB in this challenging anatomical setting.

## Data Availability

The original contributions presented in the study are included in the article/Supplementary Material, further inquiries can be directed to the corresponding author.
